# Parasite infections in a social carnivore: Evidence of their fitness consequences and factors modulating infection load

**DOI:** 10.1002/ece3.5431

**Published:** 2019-07-11

**Authors:** Susana Carolina Martins Ferreira, Heribert Hofer, Luis Madeira de Carvalho, Marion L. East

**Affiliations:** ^1^ Department of Ecological Dynamics Leibniz Institute for Zoo and Wildlife Research Berlin Germany; ^2^ Department of Veterinary Medicine Freie Universität Berlin Berlin Germany; ^3^ Department of Biology, Chemistry and Pharmacy Freie Universität Berlin Berlin Germany; ^4^ Centre for Interdisciplinary Research in Animal Health (CIISA), Faculdade de Medicina Veterinaria Universidade de Lisboa Lisbon Portugal

**Keywords:** fitness, gastrointestinal parasites, juvenile survival, resistance, Serengeti ecosystem, spotted hyena, tolerance

## Abstract

There are substantial individual differences in parasite composition and infection load in wildlife populations. Few studies have investigated the factors shaping this heterogeneity in large wild mammals or the impact of parasite infections on Darwinian fitness, particularly in juveniles. A host's parasite composition and infection load can be shaped by factors that determine contact with infective parasite stages and those that determine the host's resistance to infection, such as abiotic and social environmental factors, and age. Host–parasite interactions and synergies between coinfecting parasites may also be important. We test predictions derived from these different processes to investigate factors shaping infection loads (fecal egg/oocyte load) of two energetically costly gastrointestinal parasites: the hookworm *Ancylostoma* and the intracellular *Cystoisospora*, in juvenile spotted hyenas (*Crocuta crocuta*) in the Serengeti National Park, in Tanzania. We also assess whether parasite infections curtail survival to adulthood and longevity. *Ancylostoma and Cystoisospora* infection loads declined as the number of adult clan members increased, a result consistent with an encounter‐reduction effect whereby adults reduced encounters between juveniles and infective larvae, but were not affected by the number of juveniles in a clan. Infection loads decreased with age, possibly because active immune responses to infection improved with age. Differences in parasite load between clans possibly indicate variation in abiotic environmental factors between clan den sites. The survival of juveniles (<365 days old) to adulthood decreased with *Ancylostoma* load, increased with age, and was modulated by maternal social status. High‐ranking individuals with low *Ancylostoma* loads had a higher survivorship during the first 4 years of life than high‐ranking individuals with high *Ancylostoma* loads. These findings suggest that high infection loads with energetically costly parasites such as hookworms during early life can have negative fitness consequences.

## 1 INTRODUCTION

Parasites and their mammalian hosts have complex and dynamic relationships (Bush, Fernández, Esch, & Seed, 2001; Irvine, 2006; Knowles et al., 2013; Lello, Boag, Fenton, Stevenson, & Hudson, 2004) with long joint evolutionary histories (Hafner & Nadler, 1988). Wild mammals are typically infected with a taxonomically diverse range of gastrointestinal (GI) macroparasites and microparasites, and individuals vary considerably in their infection loads and diversity of coinfecting parasite taxa (Heitlinger, Ferreira, Thierer, Hofer, & East, 2017; Irvine, Stien, Halvorsen, Langvatn, & Albon, 2000; MacIntosh, Hernandez, & Huffman, 2010; Seltmann, Webster, Ferreira, Czirják, & Wachter, 2019). This heterogeneity is thought to be shaped by interactions between numerous factors associated with the host, the infecting parasite(s), and the environment. Disentangling the contribution of these various factors to GI parasite infection is difficult, particularly in unmanaged, wild mammalian hosts (Pedersen & Fenton, 2007; Rynkiewicz, Pedersen, & Fenton, 2015; VanderWaal & Ezenwa, 2016).

The ability of an individual to prevent, control, and clear infection is termed resistance, whereas tolerance is defined as the ability to limit the damage caused to an individual's health status and the associated Darwinian fitness cost of a given infection load (Råberg, Graham, & Read, 2009). There is growing interest in the effect of GI parasite infections on health status and components of fitness such as survival, longevity, and reproductive success in mammals (Hayward et al., 2014; Kutz, Hoberg, Nagy, Polley, & Elkin, 2004; Lynsdale, Mumby, Hayward, Mar, & Lummaa, 2017) and how phenotypic expression of both resistance and tolerance changes during an individual's life span. Most studies focus on adults, even though juveniles may be more susceptible to infection and may suffer more severe outcomes of infection than adults (Chilvers, Duignan, Robertson, Castinel, & Wilkinson, 2009; East et al., 2008; Marcus, Higgins, & Gray, 2014; Shrestha et al., 2015).

Resistance to parasite infection depends on both intrinsic and extrinsic factors (Ardia, Parmentier, & Vogel, 2011; Hayward et al., 2011; Jackson et al., 2011). In mammals, age is one intrinsic factor that can alter resistance to infection, because immune processes generally improve from early life to adulthood and then decline in old age (Simon, Hollander, & McMichael, 2015). When young, the cellular immune responses of juveniles typically differ both qualitatively and quantitatively from those of adults (Ramsburg, Tigelaar, Craft, & Hayday, 2003; Watson et al., 2016) and juveniles are less likely to have the acquired immunity that develops following exposure to pathogen antigen than adults, (Cattadori, Boag, Bjørnstad, Cornell, & Hudson, 2005; Ferreira, Torelli, et al., 2019), and thus, juveniles are generally more vulnerable to infection than adults.

The period of rapid growth and development during the juvenile life‐stage is energetically costly, and when food intake is insufficient, juveniles may resort to resource allocation trade‐offs to support growth and development at the expense of immune responses (Roff, 1992; Sheldon & Verhulst, 1996; Stearns, 1992). When this occurs, resistance to GI parasite infection should decline and infection loads increase. Furthermore, high infection loads of energetic costly parasites might compromise a host's nutritional status, thereby curtailing resource allocation to immune processes. When this leads to an increase in host susceptibility to infection, the outcome may be increased infection loads and an increase in the number of coinfecting taxa. This negative cycle might have detrimental fitness consequences such as reduced survival (Beldomenico & Begon, 2010; Beldomenico et al., 2008). Reduced survival in individuals with high infection loads indicates a lower tolerance of infection than that in animals that survive these levels of infection.

An individual's behavior may determine its chance of pathogen infection and the outcome of infection. For example, the aggregation of animals at breeding sites and water sources is thought to promote both the direct transmission of pathogens between hosts and the level of environmental contamination of such sites with infective stages (Cattadori et al., 2005; East, Kurze, Wilhelm, Benhaiem, & Hofer, 2013; Stommel, Hofer, Grobbel, & East, 2016). In social mammals, interactions between members of a group can affect the chance and outcome of infection for an individual (Duboscq, Romano, Sueur, & MacIntosh, 2016; Kappeler, Cremer, & Nunn, 2015; Nunn, Jordan, McCabe, Verdolin, & Fewell, 2015). Within‐group social status (social rank) emerges from the outcome of behavioral interactions between animals in a group, and typically, high‐ranking animals are more attractive social partners than low‐ranking animals (Seyfarth, 1977). This suggests that more frequent social interactions by high‐ranking individuals enhance the likelihood of contact with pathogens and hence they may require greater resistance against infection and/or a higher tolerance of higher infection loads than low‐ranking individuals (Marescot et al., 2018). High‐ranking individuals in mammalian groups typically have greater access to food resources and a higher nutritional status than low‐ranking individuals, and as a result, offspring reared by high‐ranking mothers are often better nourished than those reared by low‐ranking mothers (Clutton‐Brock & Huchard, 2013; Hofer, Benhaiem, Golla, & East, 2016). This suggests that juveniles reared by high‐ranking mothers (high‐ranking juveniles) should find it easier to allocate the energy and protein (Jones et al., 2011) required for the maintenance of effective immune processes and the repair of parasite damaged tissue than those reared by low‐ranking mother (low‐ranking juveniles). As immune processes and tissue repair are key components of resistance to infection, high‐ranking juveniles should experience a less severe impact of infection (for any given infection load) on their health and on fitness components such as survival than low‐ranking juveniles. Thus, high‐ranking juveniles are expected to exhibit greater tolerance of infection than low‐ranking juveniles. Several studies provide evidence that adult high‐ranking individuals do allocate more resources to immune processes and the repair of parasite damaged tissue than low‐ranking animals (Ardia et al., 2011; East et al., 2015; Flies, Mansfield, Flies, Grant, & Holekamp, 2016; Marescot et al., 2018), but studies of this kind on juveniles are rare.

A range of intestinal parasites infect wild spotted hyenas (*Crocuta crocuta*) (Baylis, 1937; Berentsen et al., 2012; East et al., 2013; Engh et al., 2003; Graber & Blanc, 1979), and recently, a metabarcoding study extended knowledge on the composition and diversity of the bacterial microbiome and eukaryome in this species (Heitlinger et al., 2017). Spotted hyenas (hereafter termed hyenas) are social carnivores that live in multifemale, multimale fission–fusion societies termed clans (Kruuk, 1972) that defend territories (Frank, 1986). In the Serengeti National Park (NP), the main prey species are migratory ungulates, and as a result of their movements, there are large fluctuations in food abundance in clan territories (Hofer & East, 1993a, 1993b). High‐ranking females have priority of access to food resources in their clan's territory and thus forage for a substantial proportion of each year in their territory. In contrast, for a substantial proportion of each year, low‐ranking females travel long distances (from 80 to 140 km per foraging trip) to forage outside their territory in areas containing large herds of migratory ungulates (Hofer & East, 1993b). The greater the distance females travel the larger the number of days between nursing visits to their dependent offspring stationed at communal dens in clan territories (Hofer & East, 1993c). Low‐ranking mothers have longer internursing intervals (Hofer et al., 2016), and their offspring have lower growth rates and are less likely to survive to adulthood than offspring of high‐ranking mothers (Hofer & East, 2003). Females reproduce throughout the year, and their offspring are dependent on milk for the first 6 months of life and are weaned between 12 and 18 months of age (Hofer et al., 2016; Hofer & East, 1993c).

Our study aims to investigate determinants of both *Ancylostoma* (nematode) and *Cystoisospora* (coccidian) infection loads in high‐ranking and low‐ranking juvenile spotted hyenas and to assess the resistance and tolerance of juveniles in these two social categories to parasite infection. We focus on *Ancylostoma* and *Cystoisospora* because both have direct life cycles that need no intermediary host, both cause damage to the epithelial lining of the small intestine, and are considered energetically costly parasites (East et al., 2015; Seguel & Gottdenker, 2017; Shrestha et al., 2015). We test predictions derived from six hypotheses: (a) the resource allocation hypothesis of life‐history theory expects high‐ranking juveniles to have lower infection loads than low‐ranking juveniles, because they should have more resources to allocate to immune processes. (b) An improvement of juvenile immune responses with age should result in a decline in infection loads with age. Higher resistance is thus expected in high‐ranking than low‐ranking juveniles and in older than younger juveniles. (c) The transmission of *Cystoisospora* by the fecal–oral route during social contacts predicts higher infection loads in high‐ranking than low‐ranking juveniles, if resistance to infection is similar in these rank categories. (d) Environmental contamination with both *Ancylostoma* and *Cystoisospora* infective stages is expected to increase as the number of clan members increases, thereby elevating infection loads. (e) The energetic cost of high infection loads of either *Ancylostoma* or *Cystoisospora* should compromise immune responses, thereby increasing the number of coinfecting taxa. (f) Finally, we expect the survival of young juvenile hyenas to adulthood and their longevity to decrease with increasing *Ancylostoma* and/or *Cystoisospora* infection loads, and the fitness cost of high infection loads to be less in high‐ranking juveniles than low‐ranking juveniles, that is, we expect high‐ranking juveniles to be more tolerant of high infection loads.

## 2 MATERIALS AND METHODS

### 2.1 Study host population

The study was conducted between March 2010 and August 2011 in three large clans that held territories at the junction between the woodland and plains in the center of the Serengeti NP. As this area is not within either the wet or dry season ranges of the migratory ungulates, females in all three clans conducted regular long distance foraging trips to locations outside their clan territory (East, Burke, Wilhelm, Greig, & Hofer, 2003; Hofer & East, 1993a). The mean number of animals (±*SEM*, plus minimum and maximum values) in these clans during the study period was 79.4 ± 0.6 adults (range 67–86 adults), 22.5 ± 0.6 juveniles (range 11–32 juveniles) in the Isiaka clan, 66.0 ± 0.6 adults (range 54–69 adults), 28.8 ± 0.4 juveniles (range 22–32 juveniles) in the Pool clan, and 81.5 ± 0.9 adults (range 67–86 adults), 20.0 ± 0.7 juveniles (range 12–28 juveniles) in the Mamba clan, respectively. These clans have been studied since May 1987, November 1989, and August 1990, respectively (East et al., 2003). Information on individual longevity used in this study was collected until the end of July 2018. Individuals were recognized by their unique spot patterns and other features (Frank, 1986; Hofer & East, 1993a). Juveniles were aged using a range of characteristics (pelage, size, locomotion) when first detected, typically within their first few weeks of life, as previously described (East et al., 2003; Golla, Hofer, & East, 1999). Sex was assessed at approximately 3 months of age using the dimorphic glans morphology of the erect clitoris or penis (Frank, Glickman, & Licht, 1991). Weaning typically occurs at 12–18 months of age (Hofer & East, 1995). Juveniles remain at the clan's communal den until approximately 12 months old where they shelter in underground burrows small enough to prevent entry of adults (Golla et al., 1999; Hofer & East, 1993c).

Juveniles obtain their social position as a result of the behavioral support they receive from their mother during interactions with other members of the clan (East et al., 2009; Smale, Frank, & Holekamp, 1993). As a result, juveniles were allocated the social rank held by their mother on the day the juvenile was sampled (Hofer & East, 2008). To construct linear dominance hierarchies for females in each clan, we recorded ad libitum submissive behaviors during dyadic interactions between adult females during frequent observation periods of approximately three hours at dawn and dusk, mostly at the clan's communal den, as previously detailed (East et al., 2003; Hofer & East, 2003). Dominance hierarchies were adjusted after each loss or recruitment of adults and when dyadic interaction data revealed that an individual had increased or fallen in rank. The social ranks held by the mothers of juveniles in this study changed little between March 2010 and August 2011. To permit the comparison of ranks held by individuals in different clans, we computed a standardized rank. This measure places the ranks within a given hierarchy evenly between the highest (standardized rank: +1) and the lowest (standardized rank: −1) rank (East et al., 2003; Goymann et al., 2001). Juveniles were then categorized as high ranking if their mother's standardized rank was equal to or above 0, and low ranking if their mother's standardized rank was below 0 (as detailed by East et al., 2015; Marescot et al., 2018). We term juveniles reared by high‐ranking mothers as high‐ranking juveniles and juveniles reared by low‐ranking mothers as low‐ranking juveniles.

### 2.2 Parasites

The GI parasite community in juveniles found by this study included helminths (*Ancylostoma*, Spirurida and *Trichuris, Dipylidium*, *Diphyllobothrium*, Taeniidae) and the apicomplexan *Cystoisospora* (Table 1). Infection with adult *Ancylostoma* hookworms is energetically costly (Seguel & Gottdenker, 2017) because adults live attached to the mucosal layer of the small intestine and feed on intestinal mucosa and blood. Adults shed eggs into the intestinal lumen which are voided in host feces (Sowemimo & Asaolu, 2008; Urquhart, Armour, Duncan, Dunn, & Jennings, 1996). Eggs hatch when environmental conditions (substrate, temperature and moisture) are suitable and larvae molt twice before they are infective. Infection occurs by ingestion or when larvae penetrate the skin of a host and then migrate through host tissue to the small intestines (Urquhart et al., 1996). Larvae can be transmitted to the offspring through the transmammary route during lactation in some host species (Burke & Roberson, 1985; Urquhart et al., 1996) or by ingestion of paratenic hosts with larvae infected tissue (Lee, Little, & Beaver, 1975). The pathogenic effects result from the blood loss instigated by adult worms and the damage adult worms cause to the intestinal epithelium (Urquhart et al., 1996).

**Table 1:** Gastrointestinal parasite taxa in 154 samples from juvenile spotted hyenas (*N* = 96) identified in fecal samples

Parasite	Phylum	Prevalence	Mean intensity	Median intensity	Mean abundance		Ratio variance/mean abundance
*Ancylostoma*	Nematoda	94.2	1,784	503	1,680	13,530	
*Dipylidium*	Platyhelminthes	59.7	–	–	–	–	
*Cystoisospora*	Apicomplexa	53.0	1,811	114	976	40,783	
*Diphyllobothrium*	Platyhelminthes	50.0	9,732	2,451	4,866	73,146	
Taeniidae	Platyhelminthes	8.4	24	16	2	83	
*Trichuris*	Nematoda	8.4	29	16	2	105	
Spirurida	Nematoda	6.5	60	16	4	296	

Note

Indicated are the percentage of infected host individuals (prevalence, %), mean and median infection loads of infected hosts (mean and median intensity, number of eggs or oocysts/g feces), mean infection load across all hosts, including the noninfected hosts with a load of 0 (mean abundance, number of eggs or oocysts/g feces) and the ratio of the variance to the mean of abundance.

*Cystoisospora* (formerly termed *Isospora*) infects epithelial cells lining the host's small intestine and is predominantly transmitted by the fecal–oral route (Lindsay, Dubey, & Blagnurn, 1997; Shrestha et al., 2015). Oocysts in feces can remain infective for weeks under favorable environmental conditions, and sporulation may take <16 hr at 30°C (Lindsay et al., 1997). Following the infection of intestinal epithelial cells, asexual reproduction occurs, thereby enhancing the infection load in these cells in relation to the infection dose. During this phase, histological changes in the small intestine occur (Lindsay et al., 1997; Shrestha et al., 2015) resulting in clinical signs such as diarrhea and weight loss (Lindsay et al., 1997; Mengel et al., 2012). Sexual reproduction occurs after a period of several to many days, depending on the species of *Cystoisospora*. Oocysts are then shed in the host's feces (Lindsay et al., 1997; Mengel et al., 2012). For details on life cycles of spotted hyena GI parasites other than *Ancylostoma* and *Cystoisospora,* please see Table S1.

### 2.3 Parasite screening

Fresh feces (*N* = 154) were immediately collected after deposition from 96 known juveniles (*N* = 50 males; *N* = 46 females) between 36 and 726 days of age (median age 188 day, mean age 214 days). Each fecal sample was mechanically mixed, and one aliquot was stored in a 4% formalin solution at room temperature until processed.

We determined fecal egg counts (FECs, eggs/g feces) and fecal oocyst counts (FOCs, oocysts/g feces) as previously described (East et al., 2015; Heitlinger et al., 2017) using the McMaster egg flotation technique following Foreyt (2001). We screened 2 g of feces per sample and used a potassium iodide (KI) solution with a specific weight of 1.5 g/ml and with a dilution ratio of 1:15 (Meyer‐Lucht & Sommer, 2005). The McMaster slide counting chambers were loaded and left for 5 min before parasite eggs/oocysts were identified, measured, and counted in four chambers per sample using a compound microscope at 100× magnification (Jenaval, Carl Zeiss). Taxa were identified by conventional morphological criteria using veterinary parasitology reference manuals (Bowman, Hendrix, & Lindsay, 2002; Eckert, Friedhoff, Zahner, & Deplazes, 2008; Foreyt, 2001; Zajac & Conboy, 2012). Taxonomic identification to the resolution of order, family, or genus level varied between taxa. Results are presented as eggs or oocysts per gram of feces (EPG or OPG). These values were calculated by dividing the total number of eggs counted by the mass of feces in the counted chambers, which corresponds to the mass of feces measured per sample (2 g) divided by the volume of KI solution used (28 ml) and multiplied by the total volume counted (0.6 ml). To investigate the reliability of detecting the presence or absence of infection at low parasite loads, we repeated the analysis using three samples with low parasite egg loads. This revealed that the chance of detecting *Dipylidium* was 33% (2 of 6 counts), *Trichuris* 50% (7/14), Taeniidae 83% (5/6), Spirurida 100% (4/4), and *Cystoisospora* 100% (4/4). In three of five taxa, there was a chance of a false‐negative result; hence, results from these three taxa should be interpreted with caution.

For *Ancylostoma duodenale*, fecundity (the number of eggs/g feces per worm) is density‐dependent, declining from 287 eggs to approximately 100 eggs for the first 100 worms and then remains largely independent thereafter (Anderson & Schad, 1985); hence, FECs are a reasonable index of adult worm infection load. Also, Heitlinger et al. (2017) found a significant positive correlation between egg or oocyst counts and the amount of parasite DNA in the feces for the taxa identified as *Ancylostoma*, *Diphyllobothrium,* and Coccidia in this study population. In pigs, *Trichuris suis* egg counts are deemed a reliable approximate estimate of the number of adult worms per host, that is, parasite load (Gassó et al., 2015). Fecal egg counts are not a reliable method to quantify parasite loads in cestodes of the order Cyclophillidea (e.g., Taeniidae) which release eggs from gravid proglottids. *Dipylidium* eggs are released inside egg packages or as gravid proglottids which creates a clumped distribution of eggs, whereas when egg packages are broken, eggs are spread in the feces (Bowman et al., 2002). To estimate infection prevalence with *Dipylidium,* we combined the presence of eggs in feces and the occurrence of *Dipylidium* proglottids on the surface of feces when collected (for details see East et al., 2013).

### 2.4 Data analysis

The prevalence of infection is the proportion of individuals infected by a particular parasite taxon. Infection load corresponds to the FEC or FOC of a particular parasite taxon per feces. Mean intensity of infection describes the mean value of infection load of a particular parasite taxon among infected hosts. Mean abundance describes the mean of infection load of a particular parasite taxon among all host examined (Bush, Lafferty, Lotz, & Shostak, 1997; Margolis, Esch, Holmes, Kuris, & Schad, 1982). The ratio of the variance to the mean abundance was calculated to assess the degree of overdispersion (Table 1).

We used infection loads for *Ancylostoma* and *Cystoisospora* and the presence or absence of coinfection with the other parasite taxa identified in our study (Table 1) as recommended by Fenton, Viney, and Lello (2010). We applied generalized mixed‐effect linear models (GLMM) using the package “lme4” (Bates et al., 2015) in which *Ancylostoma* load (Table 2) and transformed log_10_(1+) *Cystoisospora* load (Table 3) were the response variables. Because the raw data were overdispersed, we used a negative binomial distribution with a log link function (Hilbe, 2011) and confirmed that negative binomial regressions adequately accounted for overdispersion. We used individual ID as random effect to prevent pseudoreplication of 38 individuals contributing more than one fecal sample.

**Table 2:** Predictors of *Ancylostoma* load (eggs/g feces) in juvenile spotted hyenas (*N* = 154) as estimated by a mixed‐effect negative binomial regression

Fixed effect	Estimate	*SE*	CI		*z*	*p*	*df*	*G*	*p*	AIC	ΔAIC
			Low	Low							
(Intercept)	10.45	1.83	NA	NA	5.70	<0.001					
Age at sampling	−0.01	0.001	NA	NA	−5.48	<0.001	1	22.14	<0.001	2,584.6	20.1
No. of coinfecting GI parasite taxa*	0.41	0.13	NA	NA	3.22	0.001	1	10.59	0.001	2,573.1	8.6
Clan Isiaka**	1.76	0.65	NA	NA	2.73	0.01	2	8.59	0.01	2,569.1	4.6
Clan Mamba**	1.68	0.73	NA	NA	2.29	0.02					
Maternal rank (high > low)	−0.39	0.25	NA	NA	−1.55	0.12	1	2.60	0.11	2,565.1	0.6
Number of adult clan members	−0.06	0.03	NA	NA	−2.09	0.04	1	15.17	<0.001	2,577.7	13.2
Number of juvenile clan members	0.05	0.03	NA	NA	1.37	0.17	1	1.89	0.17	2,564.4	−0.1

Note

Potential predictors included age, coinfection (scored as the number of other gastrointestinal parasite taxa), clan membership, and maternal social status. Shown are the model parameter estimates, their standard errors (*SE*) in natural log units, and *z*‐values with associated *p*‐value. Positive (negative) estimates indicate that an increase in the value of the parameter increased (reduced) egg load. Also shown are the tests for the significance of each parameter using log‐likelihood ratio tests (*G*), with associated *p*‐values and the values for the Akaike information criterion (AIC) for each alternative model when the specific predictor was removed. AIC for the full model was 2,564.5.

Abbreviations: GI, gastrointestinal.

* Presence of one or more of the following taxa: *Cystoisospora*, *Diphyllobothrium*, *Dipylidium*, Taeniidae, Spirurida, *Trichuris*.

** Clan Pool was reference clan.

**Table 3:** Predictors of *Cystoisospora* load (oocysts/g feces) in juvenile spotted hyenas log10 (1+) transformed (*N* = 154) as estimated by a mixed‐effect negative binomial regression

	Estimate	*SE*	CI		*z*	*p*	*df*	*G*	*p*	AIC	ΔAIC
			Low	High							
(Intercept)	1.63	0.87	−0.12	3.39	1.86	0.06	–				
Age at sampling	−0.001	0.001	−0.003	−0.00002	−1.96	0.05	1	4.95	0.05	481.83	1.95
No. of coinfecting GI parasite taxa*	0.22	0.08	0.05	0.39	2.63	0.01	1	6.79	0.01	484.68	4.8
Clan Isiaka**	1.48	0.33	0.82	2.14	4.44	<0.001	2	18.24	<0.001	494.12	14.24
Clan Mamba**	1.34	0.37	0.61	2.10	3.61	<0.001					
Maternal rank (high > low)	0.06	0.15	−0.24	0.35	0.42	0.68	1	0.17	0.68	478.06	−1.82
Number of adult clan members	−0.04	0.01	−0.07	−0.01	−2.95	0.003	1	8.49	0.004	486.37	6.49
Number of juvenile clan members	0.02	0.02	−0.01	0.06	1.33	0.18	1	1.66	0.20	479.54	−0.34

Note

Potential predictors included age, coinfection (scored as the number of other gastrointestinal parasite taxa), clan membership, and maternal social status. Shown are the model parameter estimates, their standard errors (*SE*) in natural log units, 95% confidence limits (CI), and *z*‐values with associated *p*‐value. Positive (negative) estimates indicate that an increase in the value of the parameter increased (reduced) egg load. Also shown are the tests for the significance of each parameter using log‐likelihood ratio tests (*G*), with associated *p*‐values and the values for the Akaike information criterion (AIC) for each alternative model when the specific predictor was removed. QAIC for the full model was 479.88.

Abbreviations: GI, gastrointestinal.

* Presence of one or more of the following taxa: *Ancylostoma*, *Diphyllobothrium*, *Dipylidium*, *Taeniidae*, *Spirurida*, *Trichuris*.

** Clan Pool was reference clan.

Fixed effect predictors of parasite load were (a) clan membership—an index of environmental variability in terms of abiotic conditions in the vicinity of clan communal den sites, which may affect the survival of parasite eggs/oocysts and infective stages (Altizer et al., 2006), (b) absolute number of juvenile clan members—an index of social contact between juveniles at the clan communal den and the deposition of juvenile feces in the vicinity of den sites, (c) absolute number of adult clan members—an index of social contact between adults and juveniles, the deposition of adult feces in the vicinity of dens, and the potential for these factors to influence parasite transmission to juveniles (see, East et al., 2013; Olarte‐Castillo et al., 2016), (d) age at sampling for individuals with several measurements, (e) the number of GI coinfecting parasite taxa (between 0 and 6 taxa), and (f) maternal social rank (high or low)—an indicator of juvenile body condition (see Hofer et al., 2016) and contact with pathogens mediated by within‐clan social interactions (Marescot et al., 2018).

We used log‐likelihood ratio tests and the Akaike information criterion (AIC) in R (R Core Team, 2015) to check whether the complete models were superior to reduced models. Models were considered similar if differences in AIC were <2.5 and preferable if the difference exceeded 6.0 (Hilbe, 2009). The significance of each predictor variable was assessed as the marginal contribution of each parameter to the full model by subtracting from the full model the log‐likelihood ratio of a second model with each specific parameter removed and testing the difference against a chi‐square distribution with appropriate degrees of freedom (see discussions in Hilbe, 2011; Hosmer, Lemeshow, & Sturdivant, 2013). To assess the global goodness of fit, we used log‐likelihood ratio tests to compare full models with intercept‐only models. In preliminary models, we included season in terms of wet and dry season and sex as fixed effects but neither had a significant effect nor improved the models as described above.

To investigate factors influencing survival to adulthood at 2 years of age, we selected samples from juveniles younger than 12 months (364 days). This resulted in a dataset with 135 samples from 84 individuals. We used a binomial logistic regression with a logit link function calculated with the R package MASS version 7.3‐45 (Venables & Ripley, 2002) and included as predictors (a) age at sampling, (b) maternal social rank (high or low), (c) *Ancylostoma* infection load*,* (d) *Cystoisospora* infection load, (e) the number of GI coinfecting parasite taxa (between 0 and 4 taxa), and (f) an interaction between age at sampling and maternal social status. To avoid pseudoreplication, we randomly chose one sample per juvenile younger than 12 months (*N* = 84).

**Figure 1:**
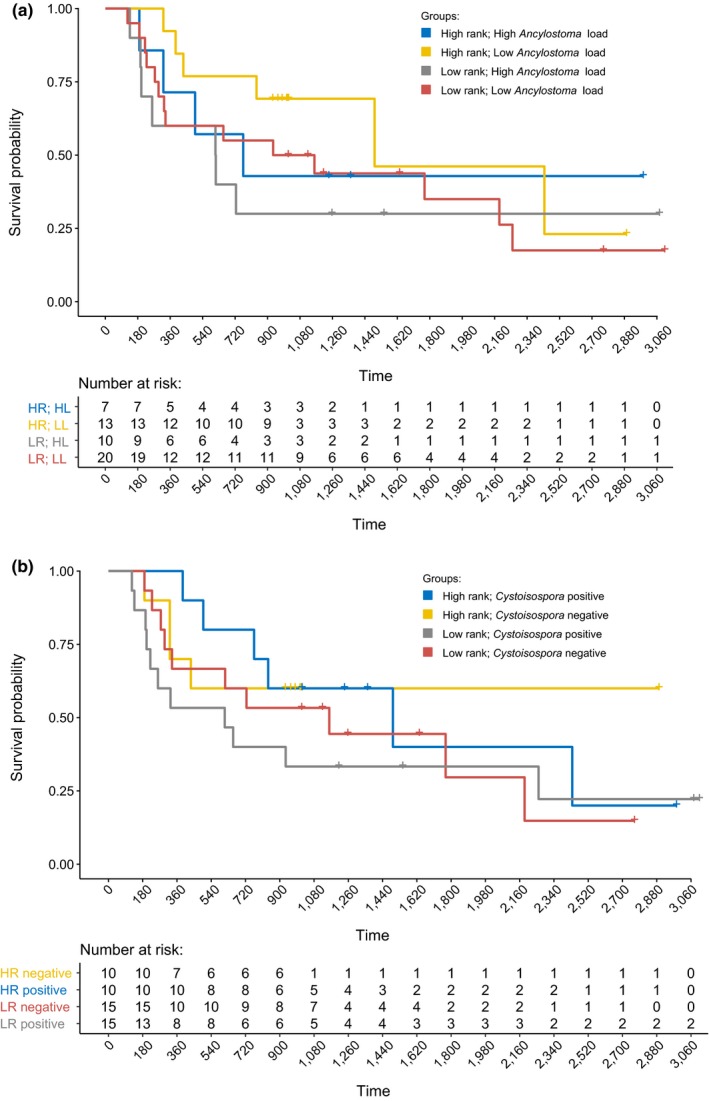
Survivorship of juvenile spotted hyenas sampled before 6 months (180 days) of age (*n* = 50). (a) Juveniles with either high (HL) or low (LL) *Ancylostoma* egg load and either a high‐ranking (HR) or low‐ranking (LR) mother; (b) juveniles either infected (positive) or not infected (negative) with Cystoisospora and either a high‐ranking (HR) or low‐ranking (LR) mother. Right‐censored measures of survival are marked as tick marks and include males that dispersed (and whose fate could not be subsequently followed), animals killed by road accidents and females that survived until the last day of observation

To investigate the tolerance of juveniles to high *Ancylostoma* infection load and to infection with *Cystoisospora,* we determined the longevity (in days) of those juveniles sampled before the age of 6 months (180 days). We applied this cutoff because juveniles <180 days old had the highest *Ancylostoma and Cystoisospora* infection loads (see results), were entirely dependent on milk, and hence, their intake of milk was likely to be sensitive to maternal rank‐related access to food and the period in days between maternal nursing visits (Hofer & East, 2003, Hofer et al., 2016). Longevity was scored as an exact value if the death date was known or as a right‐censored value (a) at the age of dispersal for males that dispersed (and whose fate could not be subsequently followed), (b) at their age on the day of the road accident for individuals killed in road accidents, and (c) the age reached by females on the last day of observation for this study (31 July 2018) if they were still alive on that day. We used Kaplan–Meier survivorship functions to construct survivorship curves for maternal rank (high and low) and infection intensity (high considered above and low below mean values of *Ancylostoma* load, Figure 1a). Similarly, for *Cystoisospora,* we constructed survivorship curves for maternal rank (high and low) but chose infection status (positive and negative) rather than infection load to improve sample size, as only 9 out of 50 individuals had infections loads above the mean, whereas 25 out of 50 individuals were scored as infected. We excluded duplicates resulting from repeated samples from the same individual that were placed in the same categories. The dataset for the *Ancylostoma* and maternal rank survivorship functions had 5 individuals sampled twice with different *Ancylostoma* load categories. The dataset for the *Cystoisospora* infection status and maternal rank survivorship function had 8 individuals sampled twice with different *Cystoisospora* infection status categories. To avoid pseudoreplication, we chose one sample per individual randomly. This dataset contained 50 juveniles.

This survival analysis was conducted using the R package survival v.2.38 (Therneau, 2015).

In all statistical models, the significance threshold was fixed at 5% and all tests were two‐tailed. All statistical analyses were performed using R version 3.2.2 (R Core Team, 2015). Unless otherwise indicated, results are presented as means ± *SD*

## 3 RESULTS

**Figure 2:**
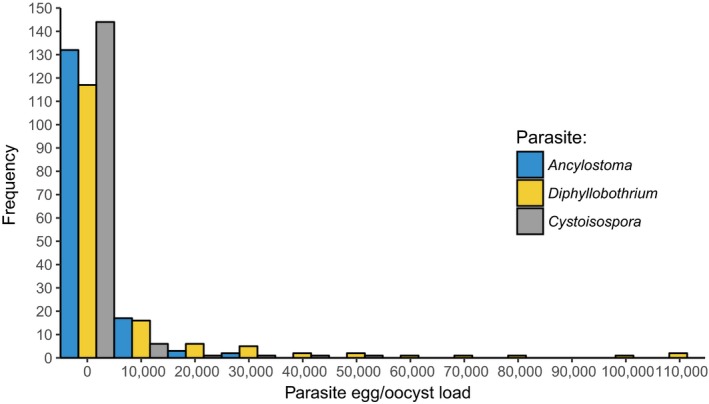
Frequency distributions of *Ancylostoma*, *Cystoisospora,* and *Diphyllobothrium* fecal egg or oocyst load (eggs or oocysts/g feces) in juvenile spotted hyenas (*n* = 154)

**Figure 3:**
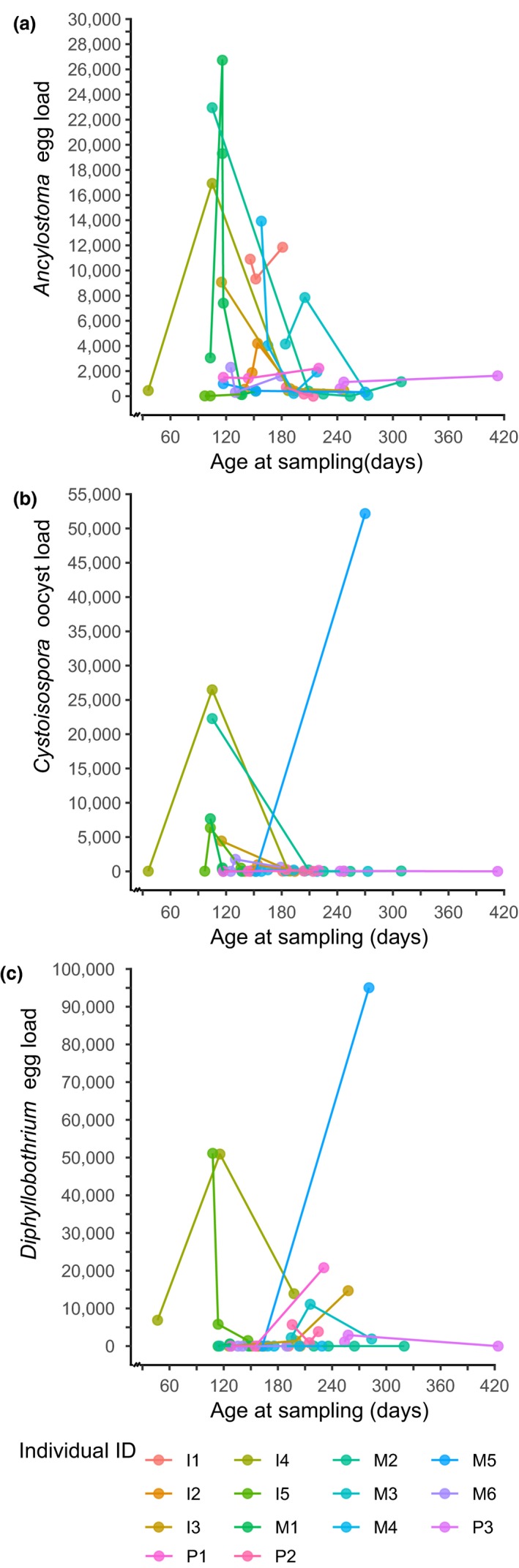
Changes in parasite infection load (eggs or oocysts/g feces) in individual juvenile spotted hyenas with (a) *Ancylostoma*, (b) *Cystoisospora*, (c) *Diphyllobothrium*

We quantified eggs or oocysts from seven parasite taxa in the feces of juvenile hyenas (Table 1). Infection prevalence was 94.2% for *Ancylostoma* and 53.9% for *Cystoisospora*. Infection loads were overdispersed (variance to mean abundance ratios > 1, Table 1) for all seven parasite taxa and are illustrated for *Ancylostoma*, *Diphyllobothrium,* and *Cystoisospora* in Figure 2. In 4 of 154 (2.6%) samples, no parasite eggs or oocysts were found. The prevalence of *Dipylidium* based on egg counts alone was 20.8% and increased to 59.7% when data on the presence of *Dipylidium* proglottids on feces at the time of collection were combined with egg count data. Changes of infection loads with age are illustrated for *Ancylostoma, Cystoisospora,* and *Diphyllobothrium* in Figure 3a–c, respectively, for all individuals sampled on at least three dates (*N* = 46 samples from 13 individuals). In general, infection load declined with age for these three most prevalent parasite genera.

**Figure 4:**
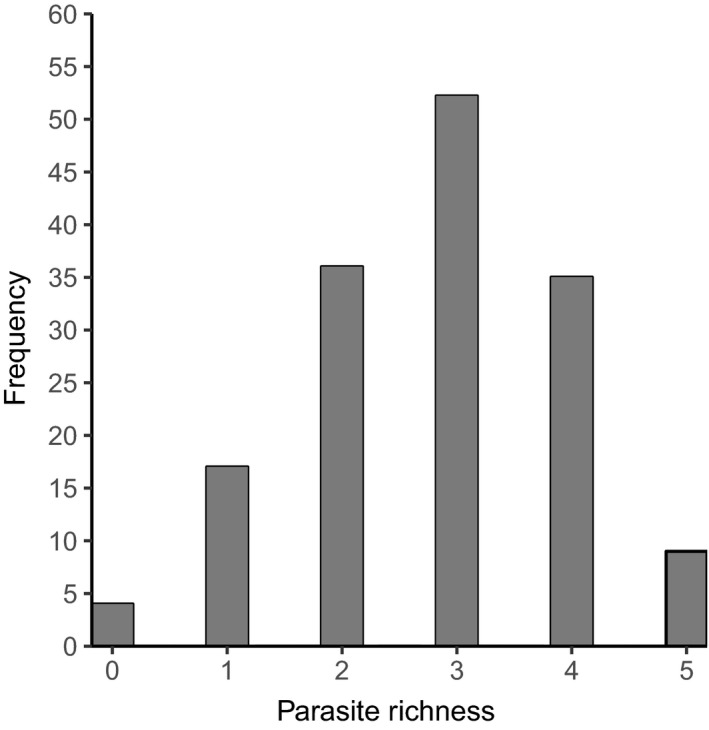
Frequency distribution of the number of gastrointestinal parasite taxa in juvenile spotted hyenas (*n* = 154)

Concurrent infection with more than one parasite taxon was frequent. Individuals were infected with 0–5 parasite taxa, the mean number of parasite taxa per individual was 2.8 ± 1.2 (*N* = 154). The frequency distribution of the number of different parasite taxa found in individual hyenas is illustrated in Figure 4.

**Figure 5:**
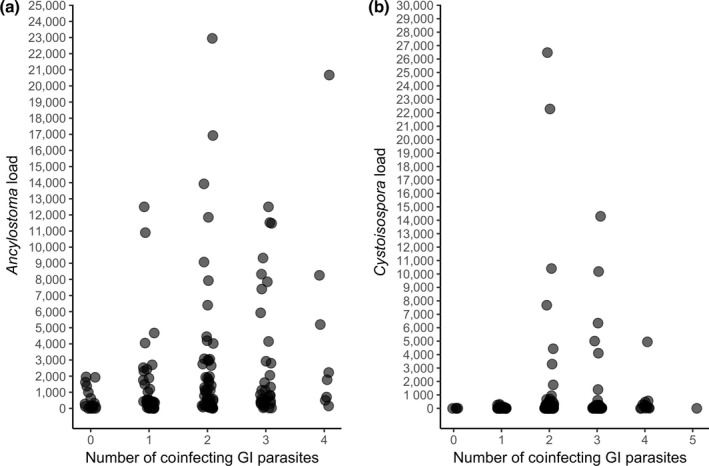
Association between the presence of different coinfecting gastrointestinal parasite taxa and (a) infection load (eggs/g feces) by *Ancylostoma*; (b) infection load (oocysts/g feces) by *Cystoisospora* (*n* = 154)

Several factors influenced *Ancylostoma* egg load (log‐likelihood ratio test *G* = 52.11, *df* = 7, *p* < 0.001, Table 2) and *Cystoisospora* oocyst load (*G* = 33.96, *df* = 7, *p* < 0.001, Table 3). *Ancylostoma* and *Cystoisospora* loads significantly decreased with host age and significantly increased with the number of coinfecting parasite taxa (Tables 2, 3, Figure 5). *Cystoisospora* load and *Ancylostoma* load significantly decreased as the number of adults in a clan increased. Both *Ancylostoma* and *Cystoisospora* loads significantly differed between clans (Tables 2, 3).

**Figure 6:**
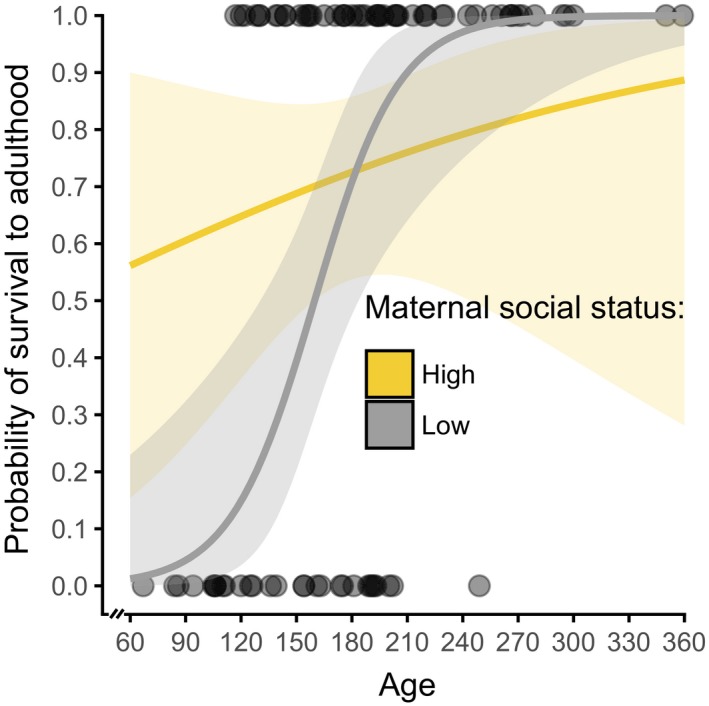
Predicted effect of juvenile age and maternal social status on the probability of juvenile survival to adulthood for spotted hyenas younger than 12 months of age with 95% confidence intervals (*n* = 84). Alongside as “dots” are the raw data of juveniles included in the model that survived (probability of survival to adulthood = 1) and did not survive (probability of survival to adulthood = 0) to adulthood

Survival to adulthood among juveniles sampled in the first 12 months of life (log‐likelihood ratio test, *G* = 33.38, *df* = 6, *p* < 0.001) decreased as *Ancylostoma* load increased but was independent of *Cystoisospora* load (Table 4). The effect of maternal social status was modulated by age. In low‐ranking juveniles, survival early in life was substantially lower than that of high‐ranking juveniles. As age increased, survival to adulthood of low‐ranking juveniles increased steeply, whereas survival to adulthood of high‐ranking juveniles increased modestly with age (Figure 6). Kaplan–Meier survivorship curves indicated that during approximately the first 4 years of life, high‐ranking juveniles with low *Ancylostoma* loads (Figure 1a) had an overall better survivorship than those with high *Ancylostoma* loads. The survivorship of low‐ranking juveniles with high *Ancylostoma* loads was mostly below that of low‐ranking juveniles with low *Ancylostoma* loads during approximately the first 5 years of life (Figure 1a). In contrast, high‐ranking juveniles infected with *Cystoisospora* had a higher survivorship until early adulthood than those that were not infected with this parasite, whereas low‐ranking juveniles that were infected with *Cystoisospora* had a lower survivorship than those not infected with *Cystoisospora* during approximately their first 5 years of life (Figure 1b).

**Table 4:** The effects of *Ancylostoma* and *Cystoisospora* load (eggs/oocysts/g feces), age, and maternal social status on survival to adulthood (at 2 years of age) for less than one‐year‐old juvenile spotted hyenas (*N* = 84)

Parameter	Regression coefficients									Odds ratios		
	Estimate	*SE*	*z*	*p*	*df*	*G*	*p*	CI		Estimate	CI	
								Lower	Upper		Lower	Upper
(Intercept)	−0.32	1.47	−0.22	0.83				−3.36	2.50	0.72	0.03	12.16
*Ancylostoma* egg load	−0.0001	0.0001	−2.07	0.04	1	4.78	0.03	−0.0003	−0.00001	1.00	1.00	1.00
*Cystoisospora* oocyst load	0.0001	0.0001	1.00	0.32	1	1.70	0.19	−0.00003	0.0003	1.00	1.00	1.00
Age at sampling	0.01	0.01	0.77	0.44	2	20.34	<0.001	−0.01	0.02	1.01	0.99	1.02
No. of coinfecting parasite taxa^a^	0.38	0.35	1.07	0.28	1	1.16	0.28	−0.31	1.09	1.46	0.74	2.97
Maternal rank: high > low	−6.86	2.83	−2.43	0.02	2	8.03	0.02	−13.20	−1.86	0.001	0.000002	0.16
Interaction age and maternal rank	0.04	0.02	2.23	0.03	1	6.38	0.01	0.01	0.07	1.04	1.01	1.08

Note

Shown are the logistic regression coefficient estimates with their standard errors (*SE*) and 95% confidence limits in natural log units as well as their conversion into odds ratios with their respective 95% confidence limits (CI), the *z*‐values and associated *p*‐values for each parameter and the results of log‐likelihood ratio tests (*G*) with associated *p*‐values. Positive (negative) estimates indicate that an increase in the value of the parameter increased (reduced) the survival to adulthood. Survival for juveniles with low maternal rank was lower than for juveniles with high maternal rank.

a Presence of one or more of the following taxa: *Diphyllobothrium*, *Dipylidium*, Taeniidae, Spirurida, *Trichuris*.

## 4 DISCUSSION

In line with other studies on parasite infections in free‐ranging wild mammals, our study on spotted hyenas revealed substantial heterogeneity between juveniles in both their *Ancylostoma* and *Cystoisospora* infection loads (Figure 2) and their number of coinfecting parasite taxa (Table 1, Figure 4). Consistent with evidence that immune processes in juvenile mammals improve with age, both *Ancylostoma* (Table 2) and *Cystoisospora* (Table 3) loads decreased as juveniles increased in age. We interpret differences in infection loads between juveniles in different clans as possible evidence that abiotic conditions at communal dens sites that influence the survival of parasite infective stages differed between clans. In contrast to our prediction, *Ancylostoma* and *Cystoisospora* loads decreased with the number of adults in a clan (Table 2). One plausible explanation could be an encounter‐reduction effect whereby adult hyenas reduce the chance of juveniles encountering *Ancylostoma* infective larvae. Also contrary to expectation, we found no evidence that *Cystoisospora* loads were affected by social interactions among juveniles (Table 3), possibly because asexual reproduction by *Cystoisospora* in infected intestinal epithelial cells contributes more to elevating infection loads in individuals than fecal–oral transmission during social interactions. We found evidence that the survival of young juveniles to adulthood (Table 4) decreased as *Ancylostoma* infection loads increased but was not affected by *Cystoisospora* infection loads (Table 4). Furthermore, the longevity of young juveniles with high *Ancylostoma* infection loads was poor compared to those with low *Ancylostoma* infection load, and this was particularly so in juveniles reared by low‐ranking mothers (Figure 1). These findings indicate that energetically costly parasites such as *Ancylostoma* can have negative fitness consequences for juveniles and that high‐ranking juveniles may be more tolerant of infection (i.e., have lower fitness costs) than low‐ranking juveniles.

Juvenile hyenas in our study population are entirely dependent on maternal milk for their growth during their first 6 months of life (Hofer et al., 2016; Hofer & East, 2003). During this early period of life, they often have their peak infection loads of *Ancylostoma*, *Cystoisospora,* and *Diphyllobothrium* (Figure 3) and hence their peak energetic costs of infection with these parasites. The survival of juveniles to adulthood and their longevity were the two components of Darwinian fitness we used to investigate the fitness consequences of *Ancylostoma* infection loads in juvenile hyenas. Our analyses revealed evidence that survival to adulthood decreased as *Ancylostoma* infection loads increased (Table 4) and that high‐ranking juveniles had a higher probability of reaching adulthood than low‐ranking juveniles (Table 4, Figure 6). Furthermore, survival to adulthood increased with age and there was an interaction between juvenile age and maternal rank (Table 4). During the first 6 months of life, the probability of survival for low‐ranking juveniles was very low and for the few low‐ranking juveniles that did survive this initial period, their probability of survival was then similar to the higher survival of high‐ranking juveniles (Figure 6). We also found evidence that in both high‐ranking and low‐ranking juveniles, survivorship (longevity) appears to be less in animals with high than those with low *Ancylostoma* loads. High‐ranking juveniles with high infection loads lived longer than low‐ranking juveniles with high loads (Figure 1a). This result is based on a relatively small number of juveniles, and thus, the effect of *Ancylostoma* loads on longevity should be further investigated with a larger sample of juveniles. Our results indicate that most animals in our study were infected with *Ancylostoma*. Given this high prevalence of *Ancylostoma* infection (Table 1) and the absence of a maternal rank effect on *Ancylostoma* loads (Table 2), we interpret these results to indicate that high *Ancylostoma* loads decrease longevity. Furthermore, we interpret the lack of a maternal rank effect on *Ancylostoma* loads (Table 2) to suggest that higher milk intake and higher growth rates in high‐ranking than low‐ranking juveniles (Hofer et al., 2016; Hofer & East, 2003) do not provide evidence that high‐ranking juveniles are more resistant to infection than low‐ranking juveniles (Figure 1a), but rather that better nourished high‐ranking juveniles have a higher “tolerance” phenotype to *Ancylostoma* load than low‐ranking juveniles. This idea needs to be more rigorously tested with a larger sample of juveniles, as it is possible that, in combination with other factors, even low *Ancylostoma* loads may reduce longevity.

Previously, we have shown that offspring reared by low‐ranking mothers have lower growth rates than those reared by high‐ranking females (Hofer & East, 2003). This is because low‐ranking mothers in our study population more often travel long distances (round trips of up to 140 km) to forage outside the clan territory than high‐ranking mothers (Hofer & East, 1993c, 2003). As a result, during the first 6 months of life, when growth is fueled by milk, low‐ranking juveniles are nursed less often and overall receive less milk than high‐ranking juveniles (Hofer et al., 2016). Furthermore, juveniles in our study population with low growth rates in the first 6 months of life have a lower probability of survival to adulthood than those with high growth rates (Hofer & East, 2003). High *Ancylostoma* infection loads are energetically costly because of substantial blood loss and damage to the intestinal wall (Urquhart et al., 1996), and have been reported to retard juvenile growth, and cause anemia and malnutrition in other mammals (Sakti et al., 1999; Seguel & Gottdenker, 2017; Stoltzfus et al., 2004). This suggests that the energetic cost of high *Ancylostoma* infection loads in rapidly growing, milk‐dependent juvenile hyenas may compromise growth and thus curtail survival to adulthood. This idea is supported by evidence that pup growth in New Zealand sea lion (*Phocarctos hookeri*) is reduced by infection with the hookworm *Uncinaria* sp (Chilvers et al., 2009), and body mass, fat deposits, and fecundity in reindeer (*Rangifer tarandus platyrhynchus*) are reduced by the nematode *Ostertagia gruehneri* (Stien et al., 2002). Using all these strands of evidence, we interpret our current finding to suggest that high‐ranking juveniles are more tolerant of high *Ancylostoma* infection loads than low‐ranking juveniles, because they are generally better nourished and thus can allocate more resources to replace the blood lost to feeding adult *Ancylostoma* and to repair the damage to the intestinal wall than low‐ranking juveniles. Similarly, a study of nematode infections in female Soay sheep on the island of St Kilda (Hayward et al., 2014) reported considerable variation in weight loss in relation to a given strongyle egg load and that animals that lost weight more slowly had a higher lifetime breeding success, indicative of a greater tolerance of infection and higher fitness. Tolerance is a trait that is thought to lead to a higher infection prevalence of a parasite in a host population and this should promote the persistence of infection (Roy & Kirchner, 2000). Consistent with this idea, our results from juvenile hyenas and those of a previous study (East et al., 2015) indicate both a high prevalence and persistence of *Ancylostoma* infection in our study clans.

Although we found no evidence that high *Cystoisospora* infection loads decreased the survival of juvenile hyenas to adulthood (Table 4), the survival probability for low‐ranking individuals that were infected with *Cystoisospora* when they were juveniles was lower throughout the juvenile period and into early adulthood than that of low‐ranking juveniles not infected by *Cystoisospora* (Figure 1b). Our study does not assess infection loads during the first few weeks after birth when we are unable to obtain feces from cubs; hence, we cannot rule out the possibility that infection during this period might decrease survival. Infection with *Cystoisospora suis* within days of birth of domestic piglets can cause severe pathologies, increased mortality and poor weight gain (Koudela & Kučerová, 1999; Lindsay et al., 1997; Shrestha et al., 2015), and an enhanced chance of coinfection (Mengel et al., 2012). Furthermore, our analysis does not distinguish between juvenile hyenas with chronic *Cystoisospora* infections that may curtail juvenile survival to adulthood from those with relatively short‐term, high infection loads that may have a limited effect on juvenile survival (Figure 3b). A preponderance of such short‐term infections in our sample may contribute to the lack of a significant effect of high oocyst infection load on juvenile survival to adulthood.

Our analysis found no evidence for the expected positive relationship between *Cystoisospora* loads in juveniles and the number of juveniles in a clan (Table 3). This relationship was expected because *Cystoisospora* is transmitted by the fecal–oral route and social interactions, particularly greeting ceremonies between young juveniles, in which participants sniff and lick their partner's anal genital region (East, Hofer, & Wickler, 1993), might be expected to increase with the number juveniles in a clan (Olarte‐Castillo et al., 2016). Environmental contamination of communal den sites might also be expected to increase with the number of juveniles, thereby raising the chance of inadvertent ingestion of oocysts by juveniles. We studied three clans that contained roughly a similar mean number of juveniles (between 20 and 29 juveniles). It is possible that the inclusion of a larger number of clans that differed substantially in the number of juveniles they contained might yield results that differ from ours.

**Figure 7:**
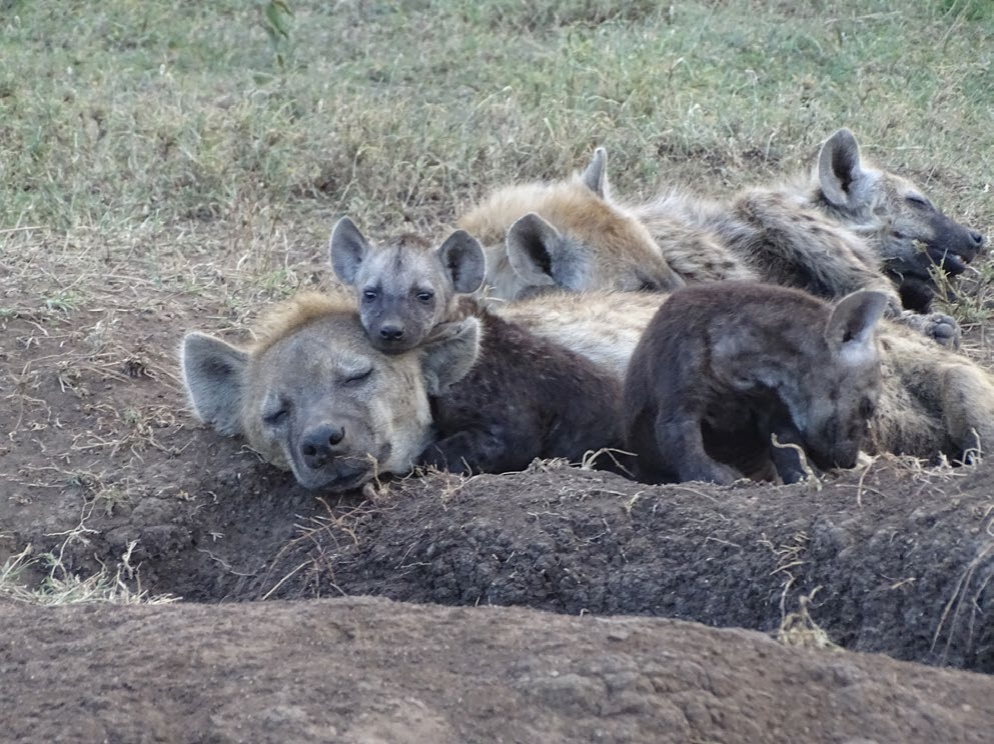
Spotted hyenas at the communal den

Our results revealed that heterogeneity in *Cystoisospora* oocyst loads shed by juveniles was considerable (Figures 2, 3b) and that high infection loads occurred mostly early in life (Figure 3b) and declined as juvenile age increases (Table 3). These findings suggest that at any given time, high infection loads are probably mostly apparent in a few younger juveniles in a clan. It is possible that high infection loads in these individuals may be linked to rapid asexual reproduction of the parasite within the intestinal epithelial cells of some individuals, followed by sexual reproduction and the shedding of high oocyst loads—rather than high infection loads resulting from fecal–oral transmission during social interactions or from environmental contamination. Juveniles that shed high *Cystoisospora* oocyst loads may act as “super‐shedders” for infection. “Super‐shedders” are thought to be important for disease transmission in several pathogens (Courtenay, Peters, Rogers, & Bern, 2017; Lloyd‐Smith, Schreiber, Kopp, & Getz, 2005; Paull et al., 2011). Because adult females in our study clans give birth throughout the year (East et al., 2003; Hofer & East, 1995), for a substantial proportion of each year, communal dens are likely to contain the young juveniles (Figure 3b) that typically shed high oocyst loads. Communal dens are likely to be hot spots for *Cystoisospora* transmission to susceptible individuals because both juveniles (this study) and lactating females at dens (East et al., 2015) shed oocysts in feces. Hyena communal den sites are locations of high use by both adults and juveniles (Figure 7) and thus can act as hot spots for the spread of pathogens, similar to bird feeders (Adelman, Moyers, Farine, & Hawley, 2015) and savannah waterholes (Stommel et al., 2016). Both super‐spreaders and hot spots of infection are important for the spread and maintenance of pathogens in host populations (Adelman et al., 2015; Courtenay et al., 2017; Lloyd‐Smith et al., 2005; Paull et al., 2011). Juvenile hyenas with high infection loads at communal dens may be important for the spread and maintenance of not only *Cystoisospora* and *Ancylostoma* (this study) but other parasite such as *Dipylidium* (East et al., 2013). For approximately 2 months after birth, defecation by young juveniles is stimulated by maternal grooming of their offspring's anus and mothers typically consume the feces produced (M. L. East and H. Hofer, personal observations). This maternal behavior may help limit oocyst contamination of dens.

*Ancylostoma* larvae emerge from eggs shed in feces and then molt twice before becoming infective filariform larvae that remain viable for weeks in moist and cool environmental conditions (Urquhart et al., 1996). Hyenas are infected when *Ancylostoma* infective larvae either burrow into their feet or pass from infected mothers to their offspring during nursing. We previously found that *Ancylostoma* infection loads were higher in low‐ranking than high‐ranking lactating females (East et al., 2015). These rank‐related differences in maternal infection loads might be expected to result in the passage of more infective larvae from low‐ranking than high‐ranking mothers to their offspring during nursing. However, even though *Ancylostoma* egg load is a good indicator of the infection load of adult worms in a host (Anderson & Schad, 1985), the number of adult *Ancylostoma* worms in the intestine may not reflect the number of migrating larvae in the female's tissue or the number of larvae shed in her milk, and this may explain why there was no significant effect of maternal rank on *Ancylostoma* infection load in juvenile hyenas (Table 2). In several species of sea lions, transmission of the hookworm *Uncinaria* by the transmammary route is thought to be important (Marcus et al., 2014).

The three study clans varied in the mean number of adults they contained (between 66 and 82 adults), and although the number of clans studied was small, our results indicate that infection loads of *Ancylostoma* (Table 2) and *Cystoisospora* (Table 3) decreased as the number of adult clan members increased. This suggests an encounter‐reduction effect (Côté & Poulin, 1995; Keesing, Holt, & Ostfeld, 2006; Mooring & Hart, 1992), which in multispecies host assemblages is termed a “dilution” effect. Evidence of an encounter‐reduction effect contradicts the expected positive relationship between the number of adults in a clan and infection loads in juveniles. How might the presence of adult clan members reduce *Ancylostoma* infection loads in juveniles? Firstly, adults exposed to the infective stages of both parasites at den sites become infected and then rapidly clear these infections, thereby decreasing the population of infective stages at dens. When the number of visits by adults to den areas is high, the removal of infective stages by adults may be sufficient to reduce encounters between juveniles and infective stages. Secondly, since adults deposit feces in communal latrines near dens, this might reduce the chance of juveniles encountering infective stages because the *Ancylostoma* infection load in the feces of adult females (East et al., 2015) is typically less than that in feces produced by juveniles (this study), probably because of a more effective immune response to infection by adults than juveniles. Unless encountered in a fresh condition, the feces of adult hyenas are often extremely hard and dry (Kruuk, 1972), whereas the feces of juveniles are more often moist and less hard (M. L. East, S. C. M. Ferreira, and H. Hofer, personal observations). This suggests that *Ancylostoma* larvae and *Cystoisospora* oocysts may develop and survive less well in adult than juvenile feces. These three possible encounter‐reduction effects may all contribute to the negative relationship between *Ancylostoma* and *Cystoisospora* infection loads in juveniles and the number of adults in the clan. Encounter‐reduction effects have been reported for a range of pathogens, including the bacterium *Mycobacterium bovis* (Huang et al., 2013; Vicente, Delahay, Walker, & Cheeseman, 2007), and both the cestode *Dipylidium* (East et al., 2013) and the enteric *Sapovirus* (Olarte‐Castillo et al., 2016) in our study population.

In line with the findings of previous studies that provide evidence that immunocompetence in juvenile mammals improves with age (Koudela & Kučerová, 1999; Ramsburg et al., 2003; Simon et al., 2015), both *Ancylostoma* and *Cystoisospora* infection loads in juvenile hyenas decreased with age (Tables 2, 3). This age effect was also apparent for infection with *Ancylostoma, Cystoisospora,* and *Diphyllobothrium* in juvenile hyenas sampled several times during their development (Figure 3a–c). The less developed immune responses of neonates develop during the juvenile period into the fully mature immune system of adults. For example, during early life, juveniles are generally more susceptible to infection by pathogens than adults, partly because of their limited capacity to mount an effective immune responses due to qualitative differences in the T‐cell populations of juveniles and adults (Ramsburg et al., 2003), and naïve, mostly young animals that lack exposure to pathogen antigens have not developed the acquired immune responses that develop as a result of exposure (Cattadori et al., 2005; Koudela & Kučerová, 1999). The decrease in *Ancylostoma* and *Cystoisospora* infection loads in juvenile hyenas with age may be the combined outcome of increased immunocompetence due to active immune responses induced by parasite exposure, which is likely to increase with age (East et al., 2001; Ferreira, Torelli, et al., 2019) and the general maturation of the mammalian immune system in juvenile hyenas with increasing age. Currently, little is known about factors that affect immunocompetence in juvenile hyenas, so in addition to age, factors such as genotype and gene expression (Gulland, Albon, Pemberton, Moorcroft, & Clutton‐Brock, 1993; Jackson et al., 2011), allostatic load, physiological processes, behavior, and diet may be relevant (Ardia et al., 2011; VanderWaal & Ezenwa, 2016).

Our results revealed a significant effect of clan membership on both *Ancylostoma* (Table 2) and *Cystoisospora* (Table 3) infection loads, with juveniles in the Isiaka and Mamba clans having higher infection loads than those in the Pool clan. This finding suggests that the survival of eggs, oocysts, and infective larvae at den sites in the Pool clan territory was lower than those in the Mamba and Isiaka clan territories, possibly because of abiotic conditions that reduce survival, such as high ambient temperatures and low soil moisture (Turner, Versfeld, Kilian, & Getz, 2012; Urquhart et al., 1996). Clan differences in infection loads may also result from behavioral differences between juveniles in the Pool and other two clans. For example, juveniles in the Pool clan may have more often changed the location of the latrines they used than those in the other two clans, thereby avoiding latrines highly contaminated with infective stages. Similarly, the use of latrines in shorter vegetation where peak daytime temperatures were higher and soil moisture was lower, might also reduce the survival of infective stages and hence infection loads.

Most wild mammal populations are concurrently infected with more than one parasite (Behnke, Gilbert, Abu‐Madi, & Lewis, 2005; Seltmann et al., 2019; Telfer et al., 2010), and this is also true for juvenile hyenas (Table 1, Figure 4). Our results revealed that as either *Ancylostoma* (Table 2, Figure 5a) or *Cystoisospora* (Table 3, Figure 5b) loads increased, the number of coinfecting GI parasite taxa hosted per juvenile also increased. The explanation for the positive relationship between infection loads and coinfecting taxa is unknown and requires further research. As maternal rank category did not have the predicted effect on either *Ancylostoma* (Table 2) or *Cystoisospora* infection loads (Table 3), we lack evidence that the immune processes of low‐ranking juveniles are more often compromised by resource allocation trade‐offs than those of high‐ranking juveniles, and thus, the role of immune processes in determining the number of coinfecting parasite taxa is also unclear. Even though potential interactions between taxa in the GI parasite community of juvenile hyenas are unknown, there was a tendency for an increase in juvenile survival to adulthood with the number of coinfecting GI parasite taxa per host (Table 4). This suggests the intriguing idea that the fitness cost of a more diverse GI parasite community may be lower than one dominated by a limited number of taxa. Previously, we found that the eukaryome in high‐ranking hyenas is more diverse than that of low‐ranking animals (Heitlinger et al., 2017) which may indicate that high‐ranking animals have a more diverse, stable, and ecologically “healthy” GI community than low‐ranking animals.

Our study identifies several factors that shape heterogeneity of parasite infections among juveniles in a wild social mammal. Disentangling key environmental factors, parasite–host mechanisms, and interactions between coinfecting parasites is difficult, particularly when noninvasive methods are required, as is the case in many protected areas or in endangered species. The benefit of noninvasive methods is that they allow repeated measures of both infection loads and components of fitness throughout an individual's life span. Further research is needed to examine in detail the effect of the variation of clan size and composition on parasite infections.

## CONFLICT OF INTEREST

The authors declare that the research was conducted in the absence of any commercial or financial relationships that could be construed as a potential conflict of interest.

## AUTHOR CONTRIBUTIONS

M.L.E, S.C.M.F., and H.H. designed the initial research, M.L.E. and H.H. performed the field work, S.C.M.F. performed the laboratory work under the supervision of L.M.C., and S.C.M.F., M.L.E., and H.H. wrote the paper. All authors contributed critically to the drafts and gave final approval for publication.

## ETHICAL APPROVAL

All protocols were noninvasive and adhered to the laws and guidelines of Tanzania. Permission to conduct research in Tanzania was granted to HH, ME, and SF by the Tanzania Commission for Science and Technology through the Tanzanian Wildlife Research Institute. Permission to undertake research within the Serengeti National Park was granted by the Tanzanian National Parks Authority. The research was also approved by the Committee for Ethics and Animal Welfare of the Leibniz Institute for Zoo and Wildlife Research under the approval number 2010‐05‐02.

## Data Availability

The dataset analyzed for this study is available from the Dryad Digital Repository, https://doi.org/10.5061/dryad.5qv7v47 (Ferreira, Hofer, Madeira de Carvalho, & East, 2019).
