# Supplementary material for: Parasite infections in a social carnivore: Evidence of their fitness consequences and factors modulating infection load
Source: Ecol Evol. 2019 Jul 11;9(15):8783–99. doi: 10.1002/ece3.5431 (PMC6686355; doi:10.1002/ece3.5431)
Supplement: Supplementary file 1 [file ECE3-9-8783-s001.docx]

Supplementary material

Parasite infections in a social carnivore: evidence of their fitness consequences and factors modulating infection load

Susana Carolina Martins Ferreira^1*^, Heribert Hofer^1,2,3^, Luis Madeira de Carvalho^4^, Marion L. East^1^

**Table S1**. Details of life cycles of gastrointestinal parasites from cats and dogs found in spotted hyenas (reference: Urquhart et al., 1996)

| **Parasite** | **Life-cycle** | **Transmission** | **Details of life-cycle** | **Pathogenesis** |
| --- | --- | --- | --- | --- |
| *Ancylostoma* | Direct | Skin penetration, ingestion, transmammary, ingestion of paratenic hosts | Adults attached to the mucosal layer of the small intestine and feed on intestinal mucosa and blood . They shed eggs which are voided in host faeces. Eggs hatch when environmental conditions (substrate, temperature and moisture) are suitable and larvae moult twice before they are infective. When infection is percutaneous, the larvae migrate through the blood stream into the lungs, bronchi and trachea, molting to L4 and then are swallowed to the small intestine and moult into adults. | Mainly due to acute and chronic loss of blood caused by the adult worm attached to the intestinal mucosa. |
| *Trichuris* | direct | Faecal-oral | Development without tissue migration in the epithelium of the host’s large intestine. Eggs shed in faeces are infective in about a month, hatching only when ingested. | Normally non-symptomatic but may cause inflammation. |
| Spirurida | Indirect: intermediate host arthropods, paratenic hosts: reptiles birds and mammals | Ingestion of intermediate or paratenic host | Varies with the species and genera from this suborder | Varies with the species and genera from this suborder |
| *Dipylidium* | Indirect: intermediate host a flea or lice | Ingestion of intermediate or paratenic host | Inhabits the small intestines of the definitive mammalian host. Eggs often in proglottids voided together with faeces. Intermediate host is infected when eggs are ingested. The cysticercoid larvae mature in the abdominal cavity of intermediate hosts. Definitive hosts are infected when they ingest infected intermediate hosts. | Normally non-symptomatic but may cause discomfort due to the active crawling from the anus of the parasite segments. |
| *Diphyllobothrium* | Indirect: 2 intermediate host a copepod crustacean and a freshwater fish | Ingestion of intermediate or paratenic host | Plerocercoid larvae mature into an adult tapeworm in the definitive host. Eggs are continuously excreted by the adult gravid segments into the intestinal lumen. In the environment, eggs require water to develop into a motile ciliated coracidium which, when ingested by the first intermediate host, develops into the first larvae stage. When ingested by the second intermediate host, develops to the second larvae stage. Life-cycle is completed when the infected fish is ingested by the definite host. | Normally non-symptomatic. |
| Taeniidae | Indirect: intermediate host a mammal | Ingestion of intermediate host | Plerocercoid larvae mature into an adult tapeworm in the definitive host. | Normally non-symptomatic. |
| *Cystoisospora* | Direct | Faecal-oral | Intracellular parasite that infect epithelial cells in the small intestine. Following the infection of intestinal epithelial cells, asexual reproduction occurs, followed by a sexual phase after a period of several to many days, depending on the species of *Cystoisospora*. Oocysts are then shed in the host’s faeces, followed by sporulation. Sporulated oocysts and remain infective for weeks under favourable environmental conditions. | Mainly due to histological changes in the intestinal mucosa during asexual division. The severity is related to parasite density and the location of parasites in the intestinal mucosa. The main clinical sign are diarrhea and weight loss. Infection might be exacerbated in the presence of other co-infecting pathogens. |
